# A high body mass index strengthens the association between the time of eye rubbing and keratoconus in a Chinese population: a case control study

**DOI:** 10.1186/s12889-023-16937-5

**Published:** 2023-10-18

**Authors:** Shengwei Ren, Runqi Tu, Liyan Xu, Yuwei Gu, Qi Fan, Qing Wang, Meng Zhu, Shanshan Yin, Chenjiu Pang, Dongqing Zhao, Kaili Yang

**Affiliations:** Henan Provincial People’s Hospital, Henan Eye Hospital, Henan Eye Institute, People’s Hospital of Zhengzhou University, Henan University People’s Hospital, Zhengzhou, 450003 China

**Keywords:** Body mass index, Eye rubbing, Interaction, Keratoconus

## Abstract

**Background:**

Although body mass index (BMI) and eye rubbing are linked to an increased risk of keratoconus (KC), the interactive effect of eye rubbing and BMI on KC is largely unknown. This study aimed to evaluate the independent and interactive effects of BMI and eye rubbing on KC and to further explore the role of environmental factors on the occurrence of KC.

**Methods:**

A total of 621 individuals (291 KC patients and 330 control individuals) were enrolled in this hospital‑based study on KC patients in Central China after individuals missing BMI data were excluded. BMI was calculated as weight in kilograms divided by the square of height in meters. Data on eye rubbing was recorded through face-to-face interviews. Generalized linear regression models were used to analyze associations among BMI, eye rubbing and KC. Interaction plots were used to describe the interactive effects of BMI and eye rubbing on KC.

**Results:**

The β and 95% confidence interval (CI) were 0.923 (0.112, 1.733) (*p* = 0.026) and 3.356 (1.953, 4.759) (*p* < 0.001), respectively, for the effect of each 10 kg/m^2^ increase in BMI and each 1 min increase in eye rubbing on KC. The interaction of BMI and eye rubbing were positively correlated with KC (*p* < 0.001).

**Conclusion:**

These findings suggested that a high BMI aggravated the negative effect of eye rubbing on KC, implying that individuals with a high BMI may be more susceptible to exposure to eye rubbing, which is related to an increased risk of KC.

## Background

Keratoconus (KC) is a common degenerative eye disease that involves ectasia of the central or paracentral steeping and thinning cornea [1, 2]. A recent study showed that the prevalence of KC is 1.38 per 1000 [3], and it is worth noting that a Netherlands study suggested that the prevalence and incidence of KC were 5 times higher than those in a previous study conducted 30 years ago [4]. Additionally, KC leads to some degree of visual impairment and decreases the quality of life of the patient, which brings a heavy social and economic burden to the patient and will be an impediment to social and economic development [4, 5].

Generally, KC usually occurs in puberty. It occurs bilaterally but asymmetrically in most cases [6]. Until now, the pathological mechanism of KC has not been clear. However, it is generally believed that KC is multifactorial and influenced by a combination of environmental, genetic, auto-metabolism, viral infections, lifestyle, and immunity factors [7]. Several studies have suggested that eye rubbing [3, 8], body mass index (BMI) [9], exposure to ultraviolet light [10], atopy [11], family history of the KC [12], and contact lens [13] are common risk factors for KC. In our previous study, we also showed that there is a separate positive relationship between atopy, eye rubbing and KC. In addition, there is an interactive effect between eye rubbing and atopy on KC, which suggests that the interaction of various environmental factors should be considered for the occurrence and development of KC [14].

BMI is linked to obesity, which is one of the strongest risk factors for sleep apnea and might be related to the occurrence of KC [9, 15, 16]. Until now, several limited studies have been conducted to explore the relationship between BMI and KC. Although several studies have failed to confirm that BMI is a risk factor for KC [15, 17], a nationwide study suggested an strong independent association between BMI and KC [9]. Furthermore, there is still a lack of evidence on the interactive effect of eye rubbing and BMI on KC, which are all closely related to the expression of inflammatory mediators. Thus, in this study, we aimed to assess associations of eye rubbing and BMI with KC and their interactive effect on KC using a case‒control study based on clinical data and KC patients in Central China.

## Methods

### Settings and participants

The subjects of the case‒control study were patients from the Henan Eye Hospital during 2019–2022. Details of the design of the case‒control study have been described elsewhere [14, 18, 19]. Briefly, in this study, the participants were recruited based on age (3 years) and sex. A total of 621 individuals (291 KC patients and 330 control individuals) were recruited in the present study after excluding the participants who had missing data on weight and height (n = 39). KC was diagnosed by the following criteria [20]: at least one positive sign on slit-lamp examination (Munson’s sign, corneal scar, Vogt’s striae, or Fleischer’s ring), Belin Ambrosio enhanced ectasia total deviation index value > 2.6 and an asymmetric bowtie pattern without or with skewed axes revealed by a corneal topography map. Participants scheduled for refractive surgery with corneal astigmatism < 1.50 D, corrected distance visual acuity in LogMAR ≤ 0.1, and spherical equivalent < 8.00 diopters were included in the control group.

### Estimation of covariates

Information on demographic characteristics and eye rubbing was collected through face-to-face interviews [14]. Educational levels were divided into middle school or below and high school or above groups. Occupation was classified into students and others. The time of eye use was determined based on the question of how much time did you spend using electronic products (computer, cell phone, etc.) and paper products (books, newspapers, etc.) per day in the last week? The time of eye rubbing was assessed using three questions. (1) How many days a week do you rub your eyes? (2) How many times a day do you rub your eyes? (3) How long do you rub your eyes each time? The time of eye rubbing (min/day) was evaluated using the formula: time (min) × frequency (every day) × day (every week)/7. Each subject’s height was measured using a tape measure while leaning against a calibrated wall without shoes. Each subject’s weight was measured using an Omron body fat body weight measurement device (V. BODY HBF-371, OMRON, Japan).

### Statistical analysis

Categorical and continuous variables are presented as number (percentage) and mean ± standard deviation (SD), respectively. The categorical variables and normally distributed variables between the KC and no KC groups were compared by the chi-square test and Student’s t test, respectively. Two models were developed to assess the associations among the time of eye rubbing, BMI and KC by using generalized linear models. Model 1 was unadjusted; Model 2 was adjusted for age, sex, education level, occupation, history of eye disease, history of eye surgery, history of ocular trauma, history of systemic disease, family history of KC, the time of eye use and myopia age. Interaction plots were applied to describe the interaction effects of BMI and the time of eye rubbing on KC after adjusting for age, sex, education level, occupation, history of eye disease, history of eye surgery, history of ocular trauma, history of systemic disease, family history of KC, the time of eye use and myopia age. All data were analyzed by using R version 4.0.0, and the statistical significance was set at a two-tailed *p*-value < 0.05.

## Results

### Study population characteristics

**Fig. 1:**
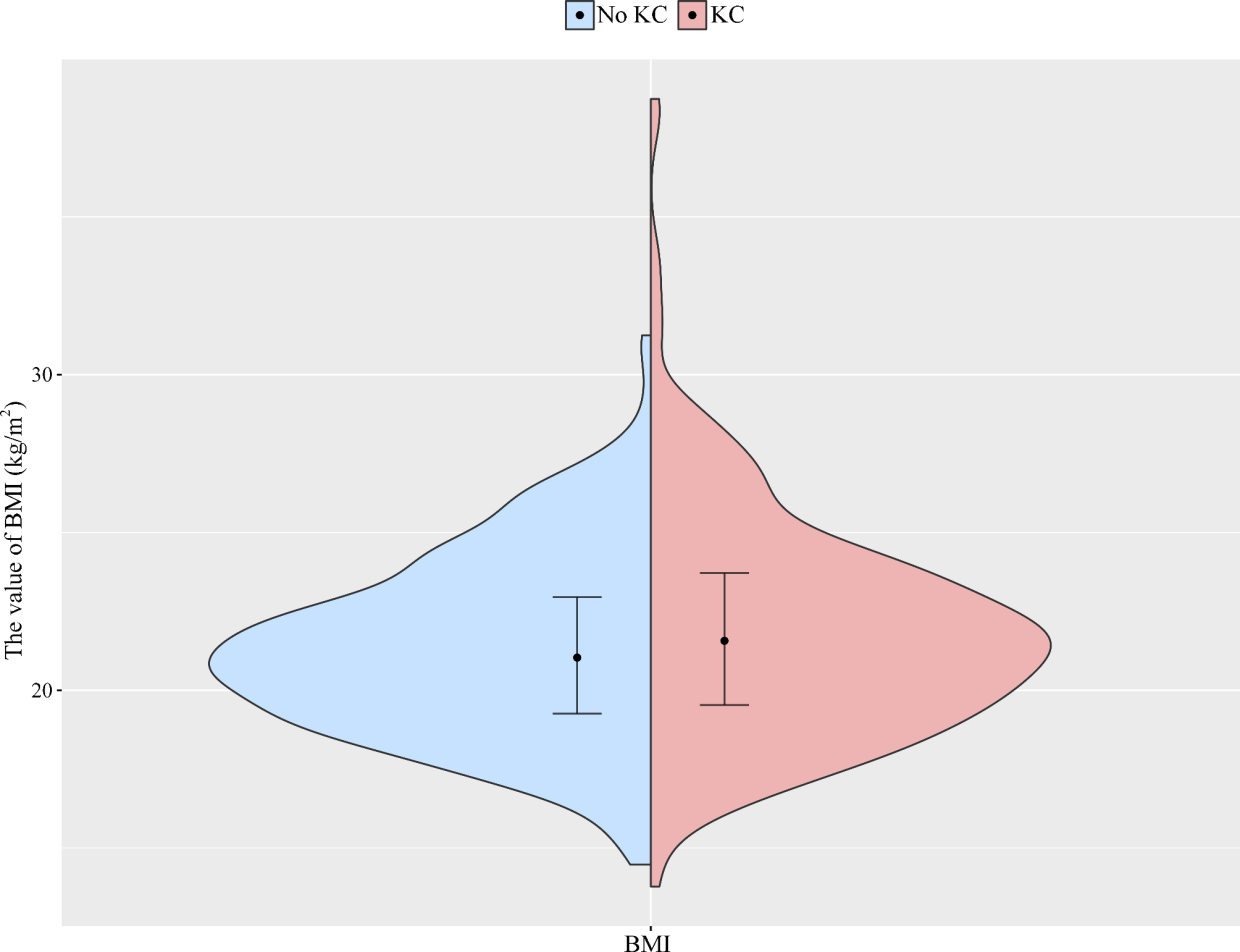
The violin plot presents the distributions of BMI (kg/m^2^)

**Fig. 2:**
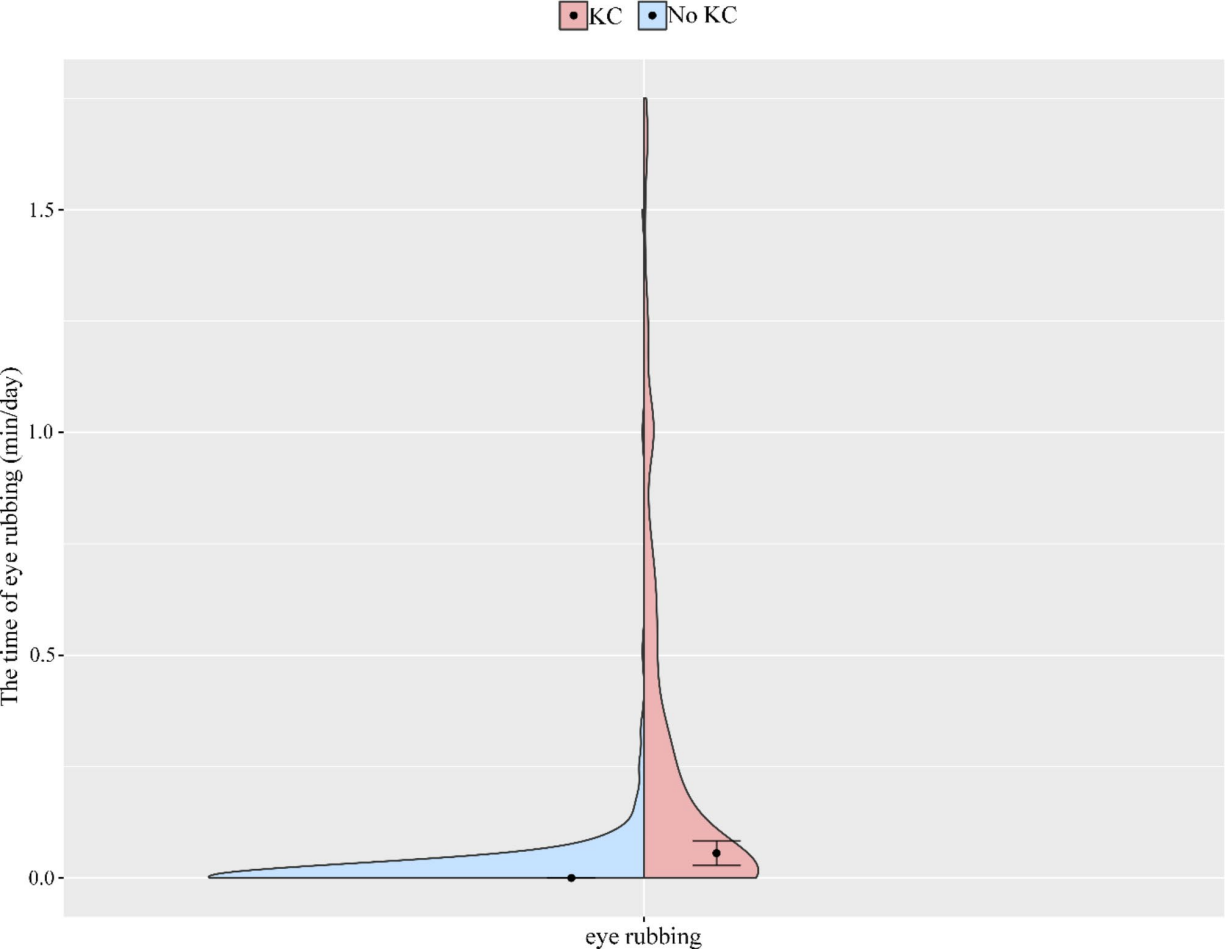
The violin plot presents the distributions of the time of eye rubbing (min/day)

Table 1 shows the demographic characteristics of the 621 subjects. The mean ± SD age of individuals with and without KC were 21.08 ± 5.58 and 20.59 ± 4.53, respectively. Different distributions of education level, occupation, history of eye disease, history of ocular trauma, history of systemic disease, myopia age, the time of eye use, BMI and the time of eye rubbing were found between individuals with and without KC (all *p* < 0.05). Figure 1 shows that the median and quartile of BMI were 21.57 (19.53–23.71) kg/m^2^ and 21.03 (19.26–22.95) kg/m^2^ in individuals with and without KC, respectively. Figure 2 shows that the median and quartile of the time of eye rubbing were 3.33 (1.67-5.00) min/day and 0.00 (0.00–0.00) min/day in individuals with and without KC, respectively.

**Table 1:** Distributions of selected variables of the study participants by different level of electronics and print-out

Variables	No KC (n = 330)	KC (n = 291)	All (n = 621)	*P*
Age (year, mean ± SD)	20.59 ± 4.53	21.08 ± 5.58	20.82 ± 5.05	0.236 ^a^
The age of KC	/	20.18 ± 5.28	/	/
Sex (n, %)				0.164 ^b^
Males	221(66.97)	210(72.16)	431(69.4)	
Females	109(33.03)	81(27.84)	190(30.6)	
Education level (n, %)				0.012 ^b^
Middle school or below	177(54.46)	127(44.1)	304(49.59)	
High school or above	148(45.54)	161(55.9)	309(50.41)	
Occupation (n, %)				< 0.001 ^b^
Student	259(78.48)	179(62.37)	438(70.99)	
Others	71(21.52)	108(37.63)	179(29.01)	
History of eye disease (n, %)				< 0.001 ^b^
No	327(99.09)	258(88.66)	585(94.2)	
Yes	3(0.91)	33(11.34)	36(5.8)	
History of eye surgery (n, %)				0.815 ^b^
No	321(97.27)	282(96.91)	603(97.1)	
Yes	9(2.73)	9(3.09)	18(2.9)	
History of ocular trauma (n, %)				< 0.001 ^b^
No	329(99.7)	275(94.5)	604(97.26)	
Yes	1(0.3)	16(5.5)	17(2.74)	
History of systemic disease (n, %)				< 0.001 ^b^
No	316(95.76)	176(60.48)	492(79.23)	
Yes	14(4.24)	115(39.52)	129(20.77)	
Family history of KC (n, %)				0.102 ^b^
No	330(100)	288(98.97)	618(99.52)	
Yes	0(0)	3(1.03)	3(0.48)	
Myopia age (year, mean ± SD)	13.13 ± 2.49	13.83 ± 2.9	13.44 ± 2.7	0.047 ^a^
The time of eye use (min, mean ± SD)	504.23 ± 233.2	549.79 ± 260.13	527.69 ± 248.27	0.027 ^a^
Hight (cm, mean ± SD)	172.86 ± 7.74	170.24 ± 8.69	171.63 ± 8.3	< 0.001 ^a^
Weight (kg, mean ± SD)	63.97 ± 11	63.79 ± 13.01	63.88 ± 11.98	0.852 ^a^
BMI (kg/m^2^, mean ± SD)	21.33 ± 2.85	21.9 ± 3.53	21.6 ± 3.2	0.022 ^a^
The time of eye rubbing (min/day, mean ± SD)	0.02 ± 0.11	0.25 ± 0.38	0.12 ± 0.29	< 0.001 ^a^

BMI: Body Mass Index; KC: keratoconus; SD: standard deviation; ^a^Student’s t test was used to compare normal distributed continuous variables between no KC and KC; ^b^A Chi-square test was used to test the distributions of categorical variables between no KC and KC

### Associations of BMI or the time of eye rubbing with KC

**Fig. 3:**
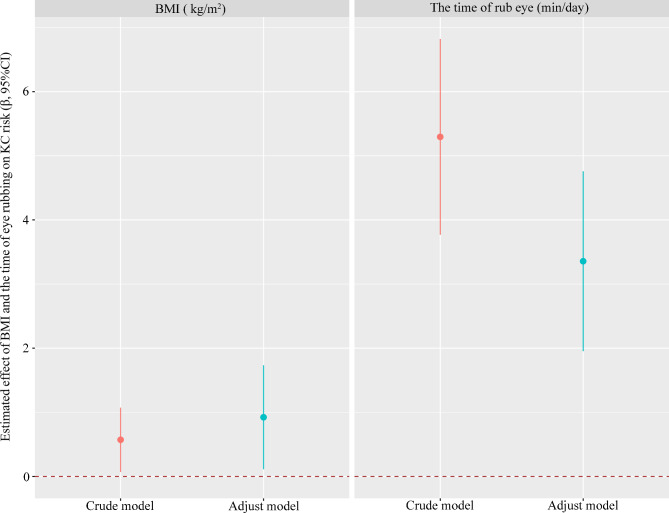
Estimated effect of BMI and the time of eye rubbing on KC risk were analyzed by generalized linear models. The adjusted model was adjusted for age, sex, education level, occupation, history of eye disease, history of eye surgery, history of ocular trauma, history of systemic disease, family history of KC, the time of eye use and myopia age. The dots and lines exhibit the β and corresponding 95% confidence interval of the associations between BMI and the time of eye rubbing and KC risk

Figure 3 shows that in the crude model, the β and 95% confidence interval (CI) of BMI and the time of eye rubbing were 0.571 (0.070, 1.073) (*p* = 0.026) and 5.295 (3.767, 6.822) (*p* < 0.001), respectively. In the adjusted model, after adjusting for age, sex, education level, occupation, history of eye disease, history of eye surgery, history of ocular trauma, history of systemic disease, family history of KC, the time of eye use and myopia age, the β (95% CI) of BMI and the time of eye rubbing were 0.923 (0.112, 1.733) (*p* = 0.026) and 3.356 (1.953, 4.759) (*p* < 0.001), respectively.

### Interactive effect of BMI and the time of eye rubbing on KC

**Fig. 4:**
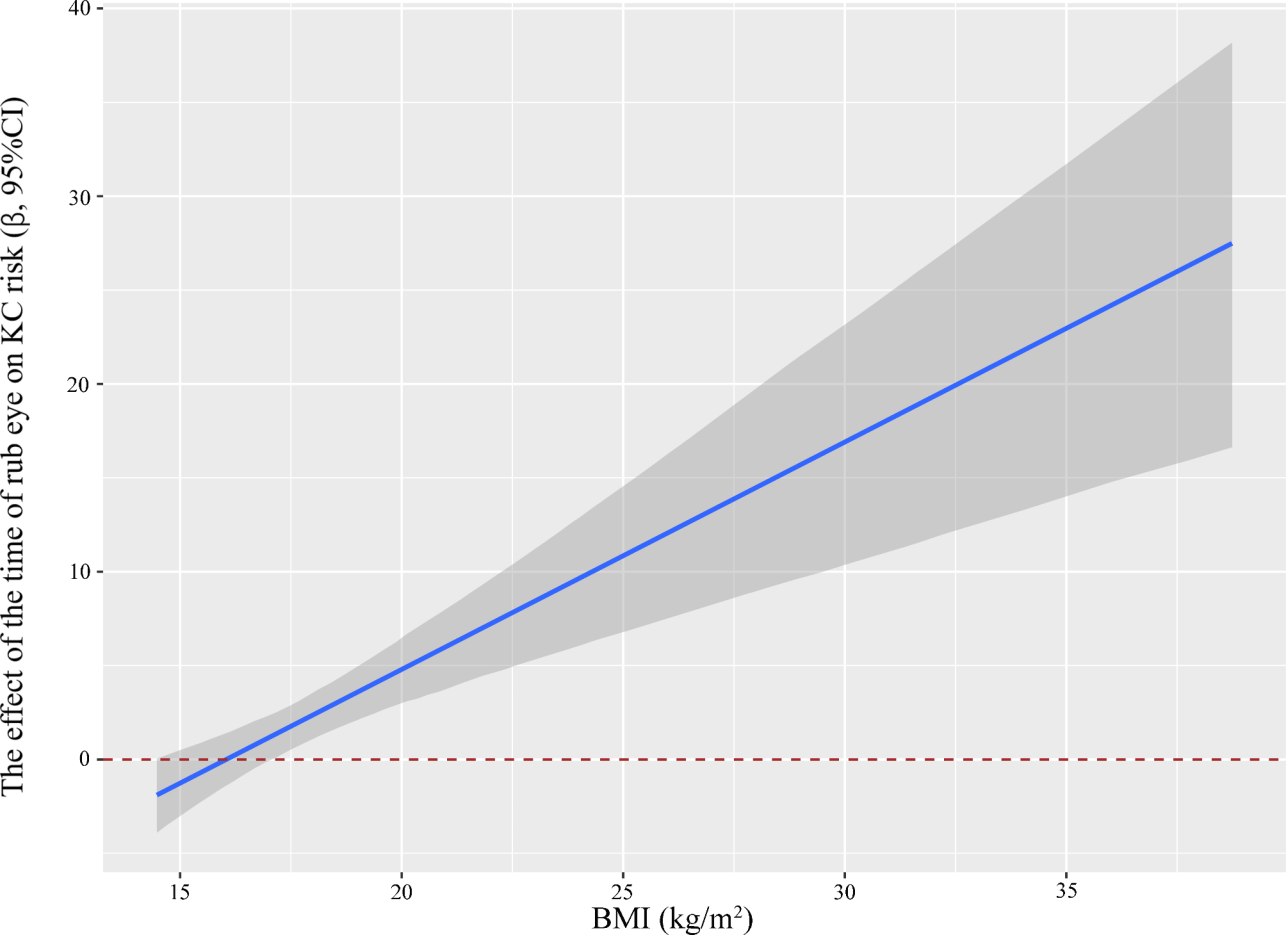
Estimated effects of the time of eye rubbing on KC risk as a function of BMI were analyzed by using generalized linear models after adjusting for age, sex, education level, occupation, history of eye disease, history of eye surgery, history of ocular trauma, history of systemic disease, family history of KC, the time of eye use and myopia age. The blue lines and grey areas represent the estimated effect and 95% confidence interval of the time of eye rubbing on KC risk along with altered values of BMI (kg/m^2^)

Figure 4 shows the estimated effect of the time of eye rubbing on KC with increasing BMI. The positive association between the time of eye rubbing and KC was strengthened as BMI increased.

## Discussion

Investigating the associations between environmental factors and KC is helpful for understanding the mechanisms of disease and is meaningful for taking early intervention measurements to avoid the occurrence of KC [3]. In the present study, BMI and the time of eye rubbing were positively associated with KC. Furthermore, the positive association between the time of eye rubbing and KC were strengthened as BMI increased.

The present study found that BMI is a risk factor for KC, which confirmed the results of a population-based cross-sectional study [9]. The study reported that obese and overweight adolescents had a higher prevalence of KC than normal weight adolescents, and the positive correlation between BMI and KC remained after adjustment for potential confounders [9]. Furthermore, a case control study in the United States reported that 50 KC patients had a high BMI value (32.4 kg/m^2^) and a higher rate of obesity (52%) than the national prevalence of 33.8% [21]. However, several studies failed to demonstrate a significant association between BMI and KC. No significant association was found between BMI and increased severity of KC in Australian patients [22]. A case‒controlled multicenter study in a Turkish population found that the mean BMI values in both the KC and control groups were within normal ranges [15]. No significant difference was found between KC and non-KC subjects in an Iran study [17]. Slater JA et al. [23] reported a ratio of 5% KC in 60 obese patients awaiting bariatric surgery, with a 100-fold increased prevalence compared with the overall population (0.05%), although the difference was not statistically significant due to the small sample size. The inconsistency of the associations between BMI and KC could be attributed to differences in race, population, study design and diagnostic criteria, and further multicenter and unified studies need to be carried out in the future.

The mechanisms underlying the association between obesity and KC are still unclear. A previous study proposed that obesity might decrease elastin in the tarsal plate, which leads to weaker eyelid skin and in turn decreases eyelid function in protecting the cornea [9]. Several studies have reported that inflammation plays roles in the occurrence of obesity and KC [24–26]. With the occurrence of obesity, the immune cells in adipose tissue infiltrate and produce inflammatory factors, such as tumor necrosis factor-α (TNF-α), interleukin (IL), and C-C motif chemokine ligand 2 (CCL2), promoting local or systemic inflammatory responses [24, 25]. These increases in inflammatory exposure in adipose tissue in the obese state also occur in the liver, pancreas, airway, brain, muscle, and possibly endothelial cells, inducing tissue damage and further leading to diabetes, asthma, liver disease, retinopathy, nephropathy, and cardiovascular disease [25, 27, 28]. This metabolic inflammatory state is chronic, with chronic maintenance of the inflammatory state without apparent resolution [25, 29]. Thus, the chronic inflammation induced by obesity may cause changes in eye inflammation, leading to chronic corneal injury, which may underlie the association between obesity and KC [26, 28, 30]. However, the hypothesis should be improved and further validated through animal studies in the future. In addition, sleep apnea may play a role in the association between obesity and KC. Obesity is regarded as one of the strongest risk factors for sleep apnea [31, 32]. Additionally, sleep apnea was also found to be significantly associated with KC [33, 34]. The collapse of the upper airways and hypoxia injuries in sleep apnea patients affect collagen tissue, corneal mechanical stability, the expression of matrix metalloproteinases (MMPs) and inflammatory mediators, which implicated in the development of KC [33–36]. The associations among obesity, KC and sleep apnea are not clearly defined, and further research is necessary.

BMI can increase the risk of KC, but the results also indicated that the time of eye rubbing was positively associated with KC. Several studies have also suggested that the longer eye rubbing times were related to an increased risk of KC [37–40]. Furthermore, another study showed that eye rubbing is a benign activity when it occurs sporadically due to eye itch and fatigue before sleep or waking. However, when it is performed too frequently or vigorously, eye rubbing becomes pathological and damages the cornea [41]. The underlying mechanisms of eye rubbing related to an increased risk for KC may involve various aspects [42]. First, persistent eye rubbing can lead to corneal trauma [43]. Second, the corneal temperature increases as a consequence of rubbing [44]. Third, rubbing-related collagen fibrils may change cone formation and corneal biomechanical stability [45]. Fourth, the damaged epithelium could cause cytokine release, myofibroblast differentiation, and changes in corneal shape and corneal biomechanical forces [46]. Finally, eye rubbing with a high frequency traumatizes keratocytes, which could lead to an inflammatory response and cause KC pathogenesis [44, 47, 48]. Eye rubbing has been widely investigated in KC, but the exact pathophysiology between eye rubbing and KC is still unclear and should be explored.

The interactive analysis showed that BMI could strengthen the association between the time of eye rubbing and KC. Obesity may lead to reduced eyelids and elastin [9], and when eye rubbing occurs, it will aggravate the damage to the cornea from eye rubbing. Moreover, the body is in a state of low-degree inflammation under conditions of obesity, which is closely related to the chronic tissue injury, and when eye rubbing causes eye inflammation, it may enhance the eye inflammatory response and further cause KC [25, 26]. In addition, the chronic habits of abnormal rubbing are relevant to corneal mechanical trauma, which triggers the release of inflammatory mediators and is accompanied by a wound healing response in keratocytes, and then facilitates cone formation [44, 48]. Both inflammation and corneal mechanical trauma were mediated in the process of eye rubbing and under conditions of obesity, which may explain the positive association between KC and the interactive effect of BMI and eye rubbing [25, 26, 30].

This case‒control study demonstrated that BMI strengthened the association between the time of eye rubbing and KC. However, several limitations should be noted. First, information about eye friction was obtained through questionnaires, which may be related to recall bias. However, all the steps were performed through standardized procedures. Second, the subjects were from the same tertiary hospital. Although patients and control individuals were matched according to some factors, extrapolation of the findings was limited and needs to be validated in a multicenter study. Third, the habit of rubbing eyes is also related to the strength and way of rubbing the eyes (knuckles and fingertips, etc.). This study only evaluated the relationship between eye rubbing time and KC. The evaluation of eye rubbing should be improved in future studies.

## Conclusion

The results indicated that the time of eye rubbing was correlated with an increased risk of KC, and those associations were strengthened by a high BMI. These findings imply that individuals with a high BMI may be more susceptible to exposure to eye rubbing, which is related to an increased risk of KC.

## Data Availability

All relevant data are within the paper. Contact to Kaili Yang (kelly1992abc@163.com) for additional information regarding data access.

## Abbreviations

BMI: Body mass index
KC: Keratoconus
CI: Confidence interval
SD: Standard deviation
TNF-α: Tumor necrosis factor-α
IL: Interleukin
CCL2: C-C motif chemokine ligand 2
MMPs: Matrix metalloproteinases
